# Breastfeeding Is Associated with a Maternal Feeding Style Low in Control from Birth

**DOI:** 10.1371/journal.pone.0054229

**Published:** 2013-01-30

**Authors:** Amy Brown, Michelle Lee

**Affiliations:** 1 Department of Public Health and Policy Studies, College of Human and Health Sciences, Swansea University, Swansea, United Kingdom; 2 Department of Psychology, College of Human and Health Sciences, Swansea University, Swansea, United Kingdom; University of Missouri-Kansas City, United States of America

## Abstract

**Background:**

The influence of maternal child-feeding style upon child weight and eating style for children over the age of twelve months is well established. However there is little empirical evidence examining maternal child-feeding style during milk feeding despite evidence that mothers who breastfeed exert lower levels of control over later diet. The aim of this paper was to examine variation in maternal child-feeding style during the first six months postpartum and to explore associations with mode of milk feeding and infant weight.

**Methods:**

The Child Feeding Questionnaire (CFQ) is frequently used to measure maternal child-feeding style in preschool children. 390 mothers with an infant aged 0–6 months completed an adapted version of the CFQ to measure maternal child-feeding style during milk feeding. Participants reported breastfeeding duration, infant weight and perceived size.

**Results:**

Principle components analysis of questionnaire items produced six factors; encouraging feeding, feeding to a routine, limiting intake, concern for weight, monitoring and perceived responsibility. Breastfeeding was associated with lower levels of control compared to formula feeding. Infant birth weight was significantly inversely associated with concern for weight, monitoring and encouraging feeding.

**Discussion:**

Formula feeding is associated with greater maternal control of child-feeding from birth whilst a lower birth weight is linked to concerns for infant weight and pressure to eat. As early maternal child-feeding relationships may impact negatively upon longer term child weight and eating style, identifying variations in maternal feeding style and understanding the factors that influence this is pertinent.

## Introduction

High levels of maternal control with regard to child energy consumption and food choices have been linked to a breakdown in child appetite regulation, food refusal and to some extent child weight [1]–[3]. Although mothers may react to child weight and eating style by increasing level of control, high control has been shown to exacerbate child eating style and weight issues [4]–[6]. The majority of studies exploring the impact of maternal feeding style have examined behaviour in relation to pre-school children [2]. However, evidence suggests that early feeding practices may play an important role in the long term development of child weight gain trajectories [7].

Formula fed infants have a greater risk of overweight compared to breastfed infants [8], [9], although how this occurs is unclear. Differing energy and nutrient compositions between breast and formula milk may explain disparities [10]. Alternatively, differences in maternal feeding style between breast and formula fed infants might place formula fed infants at greater risk of overweight [11]. As feeds given during formula feeding are usually set amounts and visible, there is increased opportunity for caregiver manipulation of energy intake [12], [13]. Conversely, breastfed infants typically need to feed on infant demand to establish milk supply [14] and amount consumed cannot be viewed [15]. This variation in early control might be critical in establishing a maternal feeding style that will persist through childhood potentially affecting child weight gain trajectories [2]. Indeed, recent evidence suggests that infants who are breastfed during the first year are rated as more satiety responsive when eating solid foods during their second year, potentially due to differences in early opportunity for appetite regulation [16].

Evidence shows that mothers who breastfeed during the first year have been shown to exert lower levels of control when feeding their child a diet of solid foods [17]–[20]. However, despite this and a recognition that maternal feeding style may vary between breast and formula fed infants, few studies have explored maternal feeding style during milk feeding. In a previous retrospective questionnaire study [11] we found that mothers who formula fed were significantly more likely to recall controlling behaviours during milk feeding; feeding to a routine and pressuring the infant to consume more milk compared to mothers who breastfed. In this current study we aim to develop a questionnaire examining maternal current behaviors during milk feeding and to explore the relationship between controlling behaviors and breast and formula feeding.

## Patients and Methods

### Participants

All participants gave informed consent prior to inclusion in this study. All aspects of this study have been performed in accordance with the 1964 Declaration of Helsinki. Approval for this study was granted by a Department of Psychology Research Ethics Committee.

Three hundred and ninety mothers with an infant aged between 0 and 6 months completed the questionnaire. Participants were recruited from local mother and baby groups and through online parenting forums based in the UK. The groups were located in areas with varying degrees of social deprivation as measured by the Welsh Indices of Multiple Deprivation [21]. Exclusion criteria included multiple birth, low birth weight (<2500 g) or premature birth (<37 weeks) [22].

For the groups, contact was made with group leaders who distributed questionnaires to group members. Questionnaires were returned to the leader in a sealed envelope or via post to the researcher. In addition posters were placed in centres around the city asking participants to contact the researcher for further details via email, phone or post. Questionnaires had information letters attached with details of how to contact the researcher if further information was required.

Study adverts were also placed on specific research request boards on online message boards on parenting forums based in the UK (e.g. www.mumsnet.com; www.bounty.com) with an online link to complete the questionnaire via surveymonkey. Details were given for how to contact the researcher if needed.

Participants completing the questionnaire via paper or online copy were given a written debrief at the end of the questionnaire and given researcher details to contact if they wanted further information. All participants were given instruction to contact their relevant health professional if completing the questionnaire had raised any questions or issues with regard to caring for their baby.

### Adapted Child-feeding Questionnaire

Participants reported their feeding practices in relation to infant feeding. They were advised to think about typical behaviour over the last two weeks rather than previous concerns or changes in behaviour. If the mother had introduced solid foods to their infant they were asked to think only about their behaviour in relation to milk feeding.

A self-report questionnaire to examine maternal use of control over milk feeding was used (Table 1). This was modeled on the Child Feeding Questionnaire [23] which explores maternal control over her child’s diet for children over the age of two years. Questions explore attitudes and behaviours for factors of perceived responsibility, concern about child weight, restriction, pressure to eat and monitoring. To design the current questionnaire, items relevant to milk feeding (e.g limiting amount of energy consumed) were reworded to reflect milk feeding. Many items on the original CFQ directly reflected behaviours relevant to milk feeding. However, for some items, the overall factor theme was used to adapt the items to be appropriate for feeding a younger infant. For example, whilst the CFQ asks ‘If my child says “I’m not hungry I try to get her to eat anyway’ our new item was phrased ‘If my baby falls asleep during a feed I encourage her to wake up to finish it’ which reflected similar beliefs but age appropriate questions. Response options were as the original CFQ.

**Table 1:** Infant Milk Feeding Questionnaire.

CFQ Factor	CFQ Question	Adapted Question and/or additional questions	IFQ Factor	Response options
Perceived Responsibility	1. When your child is at home how often areyou responsible for feeding her?2. How often are you responsible for deciding what your child’s portion sizes are?	1. How are often are you responsible forfeeding your baby?2. How often are you responsible for deciding how much milk your baby drinks?	Perceived Responsibility	1. Never2. Seldom3. Half of the time4. Most of the time5. Always
Concern about Child Weight	3. How concerned are you about your childbeing overweight?4. How concerned are you about your child maintaining a desirable weight?5. How concerned are you about your child becoming under weight?	3. I worry about my baby becomingoverweight4. I worry about my baby staying a desirable weight5. I worry about my baby becomingunderweight	Concern for infant weight	1. Strongly disagree2. Disagree3. Neither agree nor disagree4. Agree5. Strongly disagree
Monitoring	6. How much do you keep track of the sweetfood your child eats7. How much do you keep track of the snackfood your child eats8. How much do you keep track of the highfat food your child eats	6. It is important to get my baby weighed regularly7. I am concerned about my baby’s position on the growth charts8. It is important to monitor my baby’s weight gain9. I keep track of the amount of milk or lengthof time my baby feeds10. It is good to be able to see how much your baby is drinking11. It is important to get my baby weighed regularly	Monitoring	1. Strongly disagree2. Disagree3. Neither agree nor disagree4. Agree5. Strongly disagree
Restriction	9. I have to be sure that my child does not eat10. too many sweets11. I have to be sure that my child does noteat too many high fat foods12. I have to be sure that my child does noteat13. too much of her favourite food14. I intentionally keep some foods out of reach15. If I did not guide or regulate my child’seating, she would eat too much	12. I have to be sure that my baby does notdrink too much milk13. I try to stretch out feeds so my baby isfeeding less frequently14. My baby often wants to feed too much15. I limit my baby’s intake of milk16. I have to be sure that my baby does notdrink too much milk	Limiting Feeding	1. Strongly disagree2. Disagree3. Neither agree nor disagree4. Agree5. Strongly disagree
Encourage to eat	16. My child should always eat all of the17. food on her plate18. I have to be especially careful to makesure my child eats enough19. If my child says “I’m not hungry I try toget her to eat anyway20. If I did not guide or regulate my child’seating she would eat less than sheshould	17. My baby should finish the milk in herbottle or breastfeed for a certainlength of time18. I have to be especially sure that my baby drinks enough19. If my baby falls asleep during a feed I encourage her to wake up to finish it20. If I do not guide her my baby would drink much less than she should	Encouraging feeding	1. Strongly disagree2. Disagree3. Neither agree nor disagree4. Agree5. Strongly disagree
		21. If my baby does not seem hungry at a particular time I try to get her to feed anyway22. I worry about my baby’s intake of milk23. If my baby has fed recently but wants more I distract her before feeding her again24. I try to keep a certain amount of time between my baby’s feeds25. I follow a feeding routine for my baby26. I check the time to see if my baby needs a feed27. I like to be able to predict when my baby is next due a feed	Feeding to a routine	1. Strongly disagree2. Disagree3. Neither agree nor disagree4. Agree5. Strongly disagree

Milk feeding and feeding solid foods to older children are two different concepts. Therefore to ensure aspects of feeding unique to milk feeding were considered within the questionnaire, interviews were conducted with mothers to explore this issue. During interviews conducted with new mothers as part of a wider study examining influences on breastfeeding [11], [24], concepts of maternal control of infant feeding were discussed. Mothers were specifically asked about how they approached feeding their infant (e.g. encouraging milk intake, monitoring feeding) and asked to consider any issues or concerns they had specifically in relation to infant feeding. One theme that arose from these interviews was the concept of feeding to a routine e.g. four hourly or feeding on infant demand. This is a concept related to maternal control of feeding but that does not apply to feeding older children. Feeding to a routine is a strong influence and topic of contention throughout early parenting literature and books promoting approaches to caring for young infants [25]. Moreover, very strict scheduled feeding has been shown to have a negative impact upon milk supply [14]. Therefore items relating to use of a routine were additionally constructed and added alongside adapted questions. Overall 27 items were designed. The relationship between original CFQ factors and items, adapted questions and new factor names can be found in Table 1.

Participants provided infant birth weight and indicated whether they initiated breastfeeding for the first feed at birth (breastfed, expressed breast milk, formula), whether they were currently breastfeeding (exclusive breastfeeding, any breastfeeding, exclusive formula feeding) and any use of supplementary formula (including infant age at introduction). Mothers also indicated age of introduction of complementary foods, if relevant.

### Data Analysis

Principal components analysis using varimax rotation was carried out using SPSS v13, SPSS UK Ltd using all items on the adapted CFQ. Factors with Eigenvalues over 1 were retained. A factor loadings threshold of 0.5 was used to determine which variables should be retained. The factor scores computed were saved as regression scores and used for the data analysis as recommended by Tabachnik and Fidell [26]. Cronbach’s alpha was computed for each factor to examine internal consistency of the factors produced.

Multivariate ANCOVA were used to explore differences in maternal use of control (encouraging feeding, feeding to a routine, limiting intake, concern for weight and monitoring feeding) by feeding method at birth (breast, expressed milk or formula milk) and current feeding method (exclusive breastfeeding, mixed feeding or formula feeding) controlling for maternal age, education and return to work.

For mothers who initiated breastfeeding at birth but had stopped by the time of the questionnaire, Pearson’s r correlations were used to explore duration of breastfeeding and maternal control. Similarly for those who had introduced solid foods, the relationship between age of infant at introduction to solid foods and maternal control was explored using Pearson’s r correlations.

Pearson’s partial correlations were used to examine association between maternal control and maternal and infant weight, again controlling for maternal age and education.

## Results

The questionnaire was returned by 390 mothers. Age of infant ranged from one week to twenty six weeks (mean age 15.76 weeks (SD: 7.11)). The mean age of the respondents at childbirth was 29.4 (SD: 5.15) years, (range from 17 to 42) and the mean number of years in education was 13.2 (SD: 2.05). A wide range of respondents in terms of socioeconomic status took part. 53.6% of mothers were primiparous (Table 2).

**Table 2:** Sample distribution by Demographic Factors (N = 384).

Indicator	Group	N	%
Age	≤19	12	3.2
	20–24	52	13.6
	25–29	115	29.9
	30–34	124	32.2
	35≥	81	21.1
Education	No formal	16	4.1
	School	96	25.0
	College	111	28.9
	Higher	161	41.9
Marital Status	Married	219	57.0
	Cohabiting	130	33.8
	Single	24	6.0
	Widowed	1	0.3
Maternal occupation	Professional & managerial	92	23.9
	Skilled	121	31.5
	Unskilled	58	15.1
	Unemployed	31	8.1
	Stay at home parent	82	21.3

Responses were discarded for mothers who did not complete all items leaving N = 384. Items were to be discarded if 90% or more of mothers gave the same response, however this did not occur for any item. Mean scores and standard deviations for each item can be found in Table 2. Principal components analysis resulted in all but three items loading onto factors. These items were removed leaving 24 items. Items that did not load highly on any factor included ‘I worry that my baby feeds too frequently’, ‘I feed my baby whenever s/he wants feeding’ and ‘How often are you responsible for feeding your baby’. No item loaded highly onto more than one factor (Table 3).

**Table 3:** Factor structure of the milk feeding questionnaire.

	Encourage feeding	Feeding routine	Limiting intake	Concern for weight	Monitor feeding	Mean score (S.D)
My baby should finish all her milk or breastfeed for a certain length of time	**.572**	.338	−.129	.449	.394	3.84 (1.39)
I have to be especially sure that my baby drinks enough	**.659**	.098	.109	.277	.215	3.15 (1.37)
If I do not guide her my baby would drink less than sheshould	**.736**	.219	.196	.188	.037	3.84 (1.42)
If my baby falls asleep during a feed I wake her up to finish it	**.686**	.189	.067	.066	.176	3.13 (1.40)
If my baby is not hungry at feeding time I try to feed heranyway	**.703**	.148	.078	.017	.307	3.30 (1.40)
I worry about my baby’s intake of milk	**.632**	.260	.193	.347	.055	3.35 (1.46)
If my baby has fed recently but wants more I try to distracther	.150	**.727**	.267	.171	.030	3.24 (1.45)
I try to keep a certain amount of time between my feeds	.090	**.615**	.010	.080	.455	2.92 (1.42)
I follow a feeding routine for my baby	.393	**.655**	.166	.024	.233	3.17 (1.45)
I check the time to see if my baby needs a feed	.030	**.700**	.245	.021	.212	2.73 (1.45)
I like to be able to predict when my baby is due a feed	.220	**.741**	.148	.196	.037	2.47 (1.94)
I have to be sure my baby does not drink too much milk	−.282	.188	−**.820**	−.051	−.042	3.80 (1.20)
I try to stretch out feeds so my baby feed less frequently	.174	.364	**.657**	.013	−.017	3.85 (1.08)
I limit my baby’s intake of milk	.079	.178	**.729**	−.032	.292	4.41 (.79)
My baby often wants to feed too much	−.282	.188	**.820**	−.188	−.042	3.76 (1.23)
If I do not guide her my baby would drink more than sheshould	.220	.214	**.802**	.096	.066	3.78 (.92)
My baby should maintain a desirable weight	.250	.090	.158	**.803**	.188	1.83 (1.19)
I worry about my baby being overweight	−.119	.188	.051	**.800**	.158	1.82 (1.19)
I worry about my baby being underweight	.357	.053	−.399	**.583**	.136	2.03 (1.24)
It is important to get my baby weighed regularly	.163	.160	.093	.093	**.832**	2.52 (1.39)
I am concerned about my baby’s position on thegrowth charts	.343	.185	−.154	−.033	**.707**	3.25 (1.44)
It is important to monitor weight gain	.134	.028	.002	.156	**.852**	2.20 (1.24)
I keep track of the amount of milk/length of timemy baby feeds	.309	.262	.198	.216	**.538**	3.00 (1.25)
It is good to be able to see how much your baby is drinking	.367	.272	.325	.327	**.805**	2.56 (1.24)
**% of variance explained**	**32.19**	**7.68**	**5.05**	**4.80**	**3.36**	
**Cronbach’s alpha**	**.839**	**.883**	**.836**	**.851**	**.818**	

Six factors were produced explaining 68.72% of the variance. The number of items loading, percentage of variance explained and Cronbach’s alpha for these items can be found in Table 3. The factors were labeled encouraging feeding, feeding to a routine, limiting intake, concern for weight, monitoring and perceived responsibility. Cronbach’s alpha was high for each factor ranging from 0.818 to 0.890. Confirmatory factor analyses were conducted on split subsets of the data by infant gender, maternal parity and maternal age (median split) resulting in similar factor structures.

Factor six, perceived responsibility was based only on one item. Originally two questions targeted this behaviour but the item ‘How often were you responsible for feeding your baby’ failed to load highly. All participants responded that they were responsible for feeding their infant at least half of the time or more. It is likely that, due to the nature of UK employment law, mothers with an infant in this age range were on maternity leave thus taking primary responsibility for feeding their infant. Indeed, only 8.9% of the sample reported that they had returned to work, with a mean age of infant in this group of 18.25 weeks (SD: 2.89). This factor was discarded leaving 24 items in the final questionnaire. The five remaining factors on the questionnaire were examined in relation to maternal demographic background, infant characteristics, breastfeeding duration and infant weight.

### Correlation between Factors

Intercorrelations between variables were examined (Table 4). All factors were significantly correlated with each other suggesting they measured similar underlying dimensions of an overarching behaviour. Although correlations were numerous, all significant correlations were between .3 and .7 suggesting significantly different factors as opposed to multicollinearity occurring. Significant Bartlett’s and Haitovsky’s tests confirmed this [27].

**Table 4:** Significant correlations between factors.

	Encourage	Limit	Routine	Monitor	Concern weight
**Encourage**	–	.385**	.596**	.515**	.585**
**Limit**	.385**	–	.444**	.317**	−.180**
**Routine**	.596**	.444**	–	.478**	−.437**
**Monitor**	.515**	.317**	.478**	–	−.598**
**Concern weight**	.585**	−.180**	−.437**	−.598**	–

* p<0.05;

** p<0.01.

### Maternal Background, Infant Age, Breastfeeding Duration and Control

Participants indicated how their infant was fed for the first feed at birth. 277 participants breastfed (71.0%), 50 gave expressed breast milk (12.8%) and 63 formula fed (16.2%). Maternal age (F (2, 385) = 19.785, p<0.001) and education (F (2, 385) = 44.303, p<0.001) differed significantly by feeding method at birth. Mothers who breastfed or gave expressed milk at birth were significantly older and had a higher level of education than mothers opting to formula feed.

Relationships between maternal age and education and factors were examined using partial correlations controlling for infant feeding method at birth and currently.

Maternal education was significantly inversely associated with encouraging feeding (Pearson’s r = −.149, p<0.01), limiting feeding (Pearson’s r = −.319, p<0.001), monitoring (Pearson’s r = −.101, p<0.05) and concern for weight (Pearson’s r = −.467, p<0.001). Maternal age was also significantly inversely associated with encouraging (Pearson’s r = −.098, p<0.05) and limiting feeding (Pearson’s r = −.220, p<0.001).

No significant association was found between infant age at time of questionnaire and any control behavior.

### Milk Feeding and Maternal Feeding Style

Using a Multivariate ANCOVA, levels of control were compared for mothers who breastfed, gave expressed milk or formula fed at birth, using maternal age and education again as covariates (Table 5). Significant differences were seen for Encouraging feeding (F (2, 378) = 13.870, p<0.001), Feeding to a routine (F (2, 378) = 10.455, p<0.001), Limiting feeding (F (2, 378) = 32.673, p<0.001), Concern for weight (F (2, 378) = 6.341, p<0.01) and monitoring (F (2, 378) = 15.770, p<0.001). Post hoc Bonferroni's tests showed that mothers who breastfed at birth reported significantly lower levels of encouraging feeding, limiting feeding, weight concerns, feeding to a routine and monitoring feeding compared to mothers who formula fed. Furthermore, mothers who gave expressed milk reported significantly higher levels of encouraging feeding, monitoring and weight concerns than mothers who either breast or formula fed at birth but significantly lower levels of limiting feeding and feeding to a routine than mothers who formula fed. No significant difference was seen in limiting feeding or feeding to a routine between mothers who formula fed or gave expressed milk at birth (all p<0.05).

**Table 5:** Level of control by feeding method showing means and standard deviations.

Timepoint	Method	Encourage	Routine	Limit	Concern	Monitor
**Birth**	Breast	2.23(.814)	2.45(.751)	2.21(.512)	1.80(.961)	2.13 (.102)
	Expressed	3.37(.819)	3.37(.611)	3.28(.598)	2.63(1.17)	3.02 (.778)
	Formula	2.94(.808)	3.50(.813)	3.33(.597)	2.12(1.11)	2.69 (.792)
**Current**	Breast	3.02(.777)	2.61(.594)	3.08(.592)	1.38(.494)	2.11 (.668)
	Mixed	3.15(.646)	3.04(.660)	3.25(.610)	1.54(.658)	2.88 (.971)
	Formula	3.92(.644)	3.55(.686)	3.69(.437)	2.37(.121)	3.41 (.906)

Current feeding behaviour was also examined in line with control using multivariate ANOVA. Levels of control for mothers who were currently exclusively breastfeeding (n = 140), mixed feeding (n = 65) or formula feeding (n = 185) were compared. No mother was currently exclusively expressing breast milk when they completed the questionnaire with 74% of those who gave expressed breast milk at birth and whose infant was now aged over six weeks had stopped giving any breast milk by this period. Significant differences between groups were seen for all control behaviours including Encouraging feeding (F (2, 378) = 10.051, p<0.001), Feeding to a routine (F (2, 378) = 22.874, p<0.001), Limiting feeding (F (2, 378) = 5.351, p<0.001), Concern for weight [F (2, 378) = 20.053, p<0.001] and monitoring (F (2, 378) = 3.650, p<0.05). Post hoc Bonferroni tests showed that mothers who were formula feeding were significantly more likely to encourage, limit and hold weight concerns than both mothers who were either breast feeding or mixed feeding. Mothers who were formula feeding were also significantly more likely to feed to a routine and monitor feeding compared to mothers who were breastfeeding. The only significant differences between mothers who were mixed feeding or exclusively breastfeeding were that mothers who were mixed feeding were significantly more likely to feed to a routine and monitor feeding (all p<0.05).

Amongst the group of mothers who initiated breastfeeding at birth but had stopped by the time of the questionnaire (N = 106), mothers who reported stopping at an earlier time point were significantly more likely to encourage feeding (Pearson’s r = −.184, p<0.05) and have concern for infant weight (Pearson’s r = −.179, p<0.05). Moreover, amongst mothers who breastfed at birth but were not exclusively breastfeeding at the time of the questionnaire (n = 175), an earlier introduction of formula was significantly associated with greater use of encouraging feeding (Pearson’s r = .353, p<0.001) and higher concern for infant weight (Pearson’s r = .229, p<0.05).

Finally, amongst mothers who had introduced solid foods at the time of data collection (n = 100), an earlier introduction of solid foods was associated with significantly higher levels of limiting feeding (Pearson’s r = .415, p<0.001), encouraging feeding (Pearson’s r = .368, p<0.001) and concern for infant weight (Pearson’s r = .341, p<0.001).

Levels of control were also examined for infant weight at birth. A higher birth weight was associated with significantly lower levels of concern for weight (Pearson’s r = −.115, p<−.05), monitoring (Pearson’s r = −.136, p<0.01) and encouraging feeding (Pearson’s r = −.121, p<0.05). These relationships were independent of both current and initial feeding method.

A key question is whether experience of breastfeeding encourages mothers to adopt a feeding style which is low in control or whether maternal desire for control drives breastfeeding duration. To explore this further, current levels of control and feeding type were examined amongst mothers with an infant two weeks old or less (n = 43) using student t tests. It is unlikely within this short period of time that experience of feeding would modify maternal control. Within this group twenty eight mothers breastfed at birth and fifteen formula fed (no mother had given expressed milk at birth). Overall, mothers who formula fed at birth were significantly more likely to report limiting (t (41) = 5.186, p<0.001), encouraging (t (41) = 2.010, p<0.05), feeding to a routine (t (41) = 4.813, p<0.001) and having weight concerns (t (41) = 2.635, p<0.01) than mothers who breastfed at birth.

## Discussion

This paper presents the development of a multidimensional questionnaire to assess maternal reported use of control during breast or formula feeding during the first six months postpartum. Six main maternal control behaviors were identified in relation to milk feeding and their relationship with breastfeeding duration and feeding method is explored. These behaviors map closely onto those identified in the Child Feeding Questionnaire developed for children consuming solid foods. This suggests common underlying control behaviors representing perhaps a general maternal disposition towards controlling child diet that is identifiable from birth. Previous research suggests that maternal control, once established, appears to be stable [17]. Given the findings that in older children high levels of maternal control can influence child eating behaviour and weight trajectories, identifying maternal use of control at this early stage may play an important role in the understanding how these early interactions might affect the development of child weight and eating styles.

Breastfeeding was associated with significantly lower levels of maternal control supporting previous work [11]. Formula feeding appeared to be associated with high levels of seemingly opposing control behaviour e.g. limiting, scheduling feeding and encouraging feeding. However, it is likely that if a mother chooses to feed to a maternal led schedule then it is likely that at feed times the infant is encouraged to consume as much milk as possible. Potentially this relationship could emerge in one of two ways. Maternal control may develop as a consequence of milk feeding. As breastfeeding typically needs to be baby-led with frequent, irregular feeding and the amount consumed cannot easily be viewed, mothers who breastfeed may develop a feeding style which is low in control whilst the regular schedule of formula feeding might encourage mothers to adopt a more rigid approach to feeding. These initial patterns may remain stable through into later solid feeding.

Conversely, maternal desire for control may be dispositional and affect breastfeeding duration. Three main findings support this. Firstly, maternal control was not associated with infant age. If maternal control develops as a consequence of feeding experience, it would be expected that levels of control would decrease within the breastfeeding group as infant age increases. Secondly, when the sample was reduced to mothers with an infant aged two weeks old or less, differences in maternal control remained between mothers who breast or formula fed at birth. It is unlikely that this short experience of milk feeding modifies level of control. Thirdly, mothers who were mixed breast and formula feeding reported higher levels of control than mothers who were exclusively breastfeeding. Experience of feeding using a bottle may increase maternal control due to greater opportunity to manipulate milk intake. However again control was not related to infant age suggesting that desire for control may drive behaviour. Mothers who wish to be able to monitor milk intake or give feeds at particular times may choose to incorporate a bottle to gain this control. Therefore, maternal control may be dispositional and instead drive decision to breast or formula feed. Mothers who desire a feeding style which allows them to exert high levels of control may choose to formula feed, or struggle with the infant led nature of breastfeeding.

However, although maternal desire for control may affect feeding choices and patterns, the data suggests that maternal experiences surrounding the birth and milk feeding might also affect the level of control a mother desires. Mothers who expressed at birth reported significantly higher levels of elements of control related to anxiety for infant weight and growth; encouraging feeding, monitoring and concern for infant weight compared to mothers who breastfed at birth. Decision to give expressed milk at birth is often driven by birth complications [28] or infant health difficulties [29] which are in turn associated with maternal anxiety for her infant [30]. Furthermore, mothers with a lower birth weight infant were significantly more likely to encourage feeding or express concern for infant weight alongside a shorter breastfeeding duration and earlier introduction to solid foods (behaviors which are been associated with the belief that the infant needs greater energy intake [31]). Maternal control may thus be dispositional but affected by events that can increase concern for infant growth and thus level of control exerted. Clearly, a longitudinal study exploring the development and origins of maternal control from birth, considering both intentions and experiences would be worthwhile.

The current findings add to the growing evidence that variations in early maternal feeding behaviour during milk feeding which are potentially driven by wider maternal factors may have an important influence on amount of energy consumed, early appetite regulation and consequently weight gain [16]–[20]. Formula fed infants have been shown to consume more energy during milk feeding throughout infancy [32], even as soon as two days postpartum [13] and to feed more quickly [33]. Prolonged bottle use is also associated with an increased risk of overweight in preschool children [34]. Clearly, something about the mechanisms of bottle feeding allows the infant to consume greater amounts of energy and be at greater risk of overweight. As infants naturally regulate intake of milk to match energy density [35], it is likely that caregiver behaviour contributes to this greater intake. Whether this is a consequence of being able to exert control over feeding patterns more easily whilst using a bottle, or through decision to use a bottle to gain higher levels of control needs further investigation.

The study does have its limitations. Firstly the sample were self selecting which may have lead to more motivated women taking part e.g. those who particularly struggled with breastfeeding or were particularly supportive of breastfeeding. Indeed, a slightly higher than average number of women who completed the survey did initiate breastfeeding at birth (84%). This compares to recent Infant Feeding Survey figures in the UK that suggests 81% of mothers breastfeed at birth [36]. However, this disparity is small and reflective of many similar samples in infant feeding research [30].

Secondly, the sample was weighted towards an older, more educated sample although due to targeted sampling in areas of higher deprivation a wider demographic spread was found. Maternal age and education were however controlled for throughout analyses. The sample was also predominantly White British in ethnic origin (92.2%). Generalisability must be approached with caution and further research should explore maternal control of milk feeding using a population based approach.

### Conclusion

The findings have important implications for understanding breastfeeding duration, the early antecedents and development of maternal control over child feeding and the interactions between these factors. Maternal control is identifiable during the period of early milk feeding and is linked to breastfeeding initiation and duration. Potentially maternal desire for control may drive both breastfeeding duration and longer term patterns of child feeding and weight gain. If so, the benefits of early interventions to target maternal attitudes, knowledge and confidence may prove worthwhile, before behaviour and weight gain trajectories become stable. A longitudinal study is clearly needed.
